# Mindfulness-based stress reduction combined with reminiscence therapy: a non-pharmacological approach for older adults with mild cognitive impairment or mild dementia due to Alzheimer’s disease

**DOI:** 10.3389/fnagi.2026.1772932

**Published:** 2026-04-20

**Authors:** Fenglan Zhang, Yanyan Wang, Danmei Lv, Lian Cai, Fang Zhou

**Affiliations:** Department of Geriatric Medicine, Zhejiang Hospital, Hangzhou, China

**Keywords:** cognitive function, dementia, mindfulness-based stress reduction, neuropsychiatric, reminiscence therapy

## Abstract

**Objectives:**

Non-pharmacological approaches are preferable to improve functional capacity and reduce emotional disorders for dementia patients with cognitive decline. This study aimed to assess the effectiveness of mindfulness-based stress reduction (MBSR) combined with reminiscence therapy (RT) on cognitive function and behavioral and psychological symptoms in older adults with mild cognitive impairment (MCI) or mild dementia due to Alzheimer’s disease (AD).

**Methods:**

A total of 114 participants were randomly assigned to receive the reminiscence therapy combined with the MBSR program (*n* = 58) and to receive the reminiscence therapy only (*n* = 56) by using a computer-generated random table. The primary outcome measure was the Alzheimer’s Disease Assessment Scale-Cognitive section (ADAS-Cog) scale for assessing cognitive impairment. The secondary outcome measures were scores of the Cornell Scale for Depression in Dementia (CSDD) scale, Neuropsychiatric Inventory (NPI) scale, and Spirituality Index of Well-Being (SIWB) scale to evaluate depressive symptoms, behavioral symptoms, and spiritual wellbeing, respectively.

**Results:**

The RT+MBSR group exhibited lower scores in the ADAS-Cog scale, CSDD scale, and NPI scale, with a higher score in the SIWB scale than the RT group post intervention and 3 months post intervention, suggesting MBSR program combined with RT could improve cognitive function, depressive, behavioral symptoms, and spiritual wellbeing of older adults with MCI or mild dementia.

**Conclusion:**

The positive effects of MBSR program combined with RT on cognitive function and neuropsychiatric symptoms among patients with MCI or mild dementia suggest the need for more widespread use of this combination for older adults in geriatric care units.

## Introduction

Dementia mainly represented by Alzheimer’s disease (AD) is the most common neuropsychiatric condition in older adults, which is clinically characterized by memory deficits, language impairment, personality changes, and a decline in global cognitive function (Oh, 2024). Mild cognitive impairment (MCI) occurs along a continuum from cognition decline due to normal aging to dementia and affects approximately 20% of people aged ≥ 60 years (Mian et al., 2024; Overton et al., 2019). The global prevalence of dementia will rise to 131.5 million by 2050 (Shah et al., 2016). With the rapid rise of the elderly population and the increase in life expectancy, China seems to have the largest population of patients with dementia or MCI, imposing massive health and economic burdens in the coming decades (Jia et al., 2020). Patients with dementia manifest behavioral and psychological symptoms (BPSD), such as irritability, aggression, depression, anxiety, apathy, sleep problems, and wandering (Watt et al., 2024). These symptoms impose heightened psychological, social, and economic burdens on their families and caregivers (Kim et al., 2021; Montesinos et al., 2024). Studies testing non-pharmacological interventions in MCI and dementia are becoming more common as they are more acceptable to some patients and less prone to side effects (Couch et al., 2020). The development and implementation of non-pharmacological interventions not only can effectively improve cognitive function people with MCI or dementia but also reduce informal caregivers’ burden (Li et al., 2021; Sun et al., 2022; van der Ploeg et al., 2025).

Reminiscence therapy (RT) is an independent nursing intervention used in the care of older people with mental and psychological problems, and it uses recollections of past events, emotions, and thoughts by means of stimuli like old photographs, music, meaningful objects, and videos, thereby enhancing positive emotions, coping skills and resilience to aging (Yan et al., 2023). For MCI care, RT has been demonstrated with improvements on cognitive function, emotional-cognitive function, and quality of life (QoL) for older adults (Huang et al., 2025; Soria-Urios et al., 2025). For dementia care, RT has been demonstrated with improvements in cognition function and QoL, a reduction in dementia-associated agitated behaviors, and an increase in subjective wellbeing (Bayram, 2024; Perez-Saez et al., 2022; Yanagida et al., 2024b). RT has been increasingly investigated in MCI or dementia care for its positive impacts on depressive symptoms, memory, and QoL in older adults with MCI or dementia, but cognitive outcomes are inconsistent (Li et al., 2020; Yanagida et al., 2024a,Yang et al., 2022). Combining group RT with other therapeutic approaches to fully determine its long-term benefits and mechanisms is required to broaden the application of RT in diverse care settings (Yanagida et al., 2024b).

The risk of developing mild dementia due to AD from its prodromal phase MCI is increased when adverse factors, such as stress and depression, are present and accumulate. Such factors likely increase the hippocampal damage central in MCI/AD etiology and lead to compensatory mechanisms failure inducing a switch toward neurodegeneration (Daulatzai, 2013). The primary aim of mindfulness-based interventions involves the management of stress and depressive symptoms, and such interventions receive much attention in the fight against MCI and AD neurodegeneration (Larouche et al., 2015). Mindfulness-based stress reduction (MBSR) integrates mindfulness meditation into clinical care aiming to enhance self-awareness and stress management, which may serve as a useful complement for risk management (Kabat-Zinn, 1982). Mindfulness practice can lead to deep relaxation in the brain and changes in electrical frequency bands associated with attention and cognitive tasks, thus improving cognitive function, depressive symptoms, sleep quality, and mentality of patients with MCI (Cai et al., 2022; Marciniak et al., 2020). Furthermore, the MBSR program has been studied for its feasibility in reducing symptoms of anxiety and depression in pre-symptomatic frontotemporal dementia mutation carriers (Poos et al., 2022). Additionally, caregivers of patients with dementia showed positive outcomes on stress, depressive symptoms, and subjective burden from the MBSR program (Cheung et al., 2020; Giulietti et al., 2024). Recently, a new integrated and multidisciplinary cognitive rehabilitation program based on mindfulness and RT has been performed in patients affected by Parkinson’s disease and MCI, showing a significant improvement on the scores of Montreal Cognitive Assessment memory sub-scale (Reitano et al., 2023). Therefore, we propose a hypothesis with expectation that MBSR combining with RT could improve cognitive function, reduce BPSD, and enhance spirituality immediately after the intervention and after a specific period of time for older adults with MCI or mild dementia.

## Materials and methods

### Participant recruitment

The prospective study included consecutive older patients with MCI or mild dementia at Zhejiang Hospital between January 2022 and August 2024. The inclusion criteria were: (i) diagnosis of MCI or mild dementia due to AD by neurologists based on the criteria proposed by the National Institute on Aging-Alzheimer’s Association (NIA-AA) (Albert et al., 2011); (ii) a screening Mini-Mental State Examination (MMSE) score of 21–30; (iii) a Clinical Dementia Rating (CDR) scale score of 0.5 or 1.0; (iv) ability to communicate in Mandarin for engaging with the intervention; (v) living in their own houses with principal caregivers (family members or hired staff) who were familiar with the patient’s situation; and (vi) aged 65 years or older. The exclusion criteria were: (i) neurodevelopmental disorders; (ii) comorbid psychiatric disorders; (iii) bedridden elder people; (iv) ongoing or planned attendance of other psychological interventions; (v) severe visual or auditory problems; or (vi) unwilling to provide written informed consent on entry to the study. Eligible patients were randomly assigned into RT+MBSR group and RT group by using a computer-generated random table. The study protocol complied with the principles of the Declaration of Helsinki and was approved by the Ethics Committee of Zhejiang Hospital. Written informed consent to participate in this study was obtained from participants or their proxy/legally authorized representatives. Caregivers included in the study also gave written informed consent to participate in this study.

### Sample size calculation

A power analysis (G*power 3.1) was performed based on a previous randomized controlled trial investigating the Alzheimer’s Disease Assessment Scale-Cognitive section (ADAS-Cog) score between two intervention groups (Rockwood et al., 1997). This trial suggested a significant difference of ADAS-Cog score after intervention (25.4 ± 2.6 vs. 23.2 ± 0.7). According to a two-tailed test with α = 0.05 and power = 0.95, a minimum of total 42 participants was required. Accounting for a 20% dropout rate, the estimated sample size was a minimum of 50 in total to maintain robust statistical power.

### Randomization and blinding

Randomization to the treatment condition was performed by an independent researcher outside the trial. A total of 120 random numbers were generated using Microsoft Excel. Each random number corresponding to one participant was written on a separate card and then placed into a sealed, opaque envelopes to ensure allocation concealment. Ultimately, 60 participants were allocated to the RT+MBSR group and 60 to the RT group at a 1:1 ratio. Blinding was maintained for both the outcome assessor and the data analyst. The assessor, unaware of group assignments, entered all collected data into an Excel spreadsheet. Data were coded and stripped of identifying information before analysis. Group allocation was only revealed after the statistical analyses were completed.

### RT contents

All eligible patients received treatment-as-usual including conventional drug treatments (eg, memantine and donepezil) and diet guidance, and RT at the site. Although RT is not a routine standard care, it has been extensively investigated for its feasibility as a part of routine care for older people with MCI or AD (Lok et al., 2019; Wang et al., 2026). Before the study, the researcher interviewed participants and their families about individual life story, past pleasant experience, special events happening in the life, and traditional festival. RT was performed in a 45- to 60-min session from 10:00 to 11:00 a.m., once a week for 8 weeks. In each session, 6 patients gathered as a subgroup received RT with guidance from the trained staff in a quiet and bright room. The RT session was conducted with 5-min introduction (introducing the topic and group rules) at the beginning of each session, 35- to 50-min reminiscence, and a 5-min wrap-up (summarizing the content and announcing the next topic) at the end of each session. The RT procedures mainly consisted of “memory trigger,” “memory evoked,” and “memory shared.” A total of 8 topics were tailored to the personal experiences and preferences of the participants according to the interview results:

1. “First meeting”: (i) Self-introduction of the psychotherapist, 2 nurses, and group members of participants; (ii) A brief introduction to RT involving activity time, location, purpose, and content; (iii) The interests and hobbies of group members of participants.
2. “My country”: (i) A 5-min video about “The Development History of the People’s Republic of China”; (ii) historical greats in their memories; (iii) The major national or social events and the difficult situations and conditions they experienced.
3. “Old time flavor of food”: (i) Recall the delicious food they loved in their childhood; (ii) Share their favorite and famous home dishes; (iii) Talk about home dishes they are good at (briefly discuss the cooking procedures).
4. “Old music”: (i) playing timeless songs loved by old adults; (ii) talk about the stories behind the songs; (iii) share their favorite songs and singers.
5. “Old movies”: (i) playing 40-min movie clip from the movie loved by old adults; (ii) talk about the stories behind the movie; (iii) share their favorite movies and actors.
6. “My family and friends”: (i) family and friend introduction according to the related photographs; (ii) talk about the happiness with their family members; (iii) talk about the happiness with their friends.
7. “Traditional festival”: (i) A 5-min video introducing Chinese traditional festival; (ii) talk about their favorite festival; (iii) discuss how to celebrate it.
8. “Personal accomplishments and goals”: (i) their dreams of youth; (ii) their accomplishments during their whole life; (iii) present or wanted interests and hobbies.

Before each session, the psychotherapist and nurses prepared enough tangible stimuli to enable the triggering of positive memories corresponding to the topic of each session. These tangible stimuli included photographs from patients’ lives, precious items, videos, old music, old films, and historical stories. The psychotherapist encouraged group members to express and share the events and experiences they remembered with the other group members, gave time to think and an equal opportunity to speak, corrected and adjusted their story to ensure the content integration of the participant’s story. If unpleasant disputes occurred, the psychotherapist promptly changed the topic to avoid arguments or conflict among group members. The fidelity of RT was ensured by the external monitoring performed by an independent researcher outside through inquiring to participants (two in random per session group) after each session. Any missing attendance or drop-outs will be followed up with a telephone call.

### MBSR program

Patients in the MBSR+RT group received additional MBSR program administered by trained and certified instructor. The MBSR program began first with the “introduction to mindfulness” session, followed by 8 weekly sessions, each lasting 2.5 h. Additionally, there was a one half-day silent retreat lasting 6 h in week 6. The “introduction to mindfulness” session included clarifying the objective of the intervention, logistics of the MBSR course, formal and informal practices, and importance of home practice. Mindfulness was cultivated through both formal (body scan meditation, sitting meditation, mindful walking and eating, mastery and pleasure activities, cognitive restructuring) and informal practices (bringing mindfulness to routine activities, including short breathing meditation, awareness of pleasant and unpleasant events with respect to feelings, thoughts and body feelings) (Marciniak et al., 2020). Participants were guided to do formal homework exercises including mindfulness meditation, breathing practices, and gentle yoga each day. More gentle movements were adapted to the patients’ physical limitations. Besides, the length of formal practices was shortened from 45 to 30 min. The fidelity of MBSR was ensured by a fixed number and length of sessions, a course manual, an external monitoring, and homework monitoring. An independent researcher outside was responsible for the external monitoring by inquiring to participants (two in random per session group) about the curriculum after each session. The homework monitoring was achieved by asking the participants to record their home practice in video, and the videos were collected and checked during the weekly sessions. Any missing attendance or drop-outs will be followed up with a telephone call.

### Outcome measures

The primary outcome measure was the ADAS-Cog scale for assessing cognitive impairment. The secondary outcome measures were scores of the Cornell Scale for Depression in Dementia (CSDD) scale, Neuropsychiatric Inventory (NPI) scale, and Spirituality Index of Well-Being (SIWB) scale to evaluate depressive symptoms, behavioral symptoms, and spiritual wellbeing, respectively. Outcome measures were collected by researchers through face-to-face interviews with patients and their long-term caregivers for informant ratings. Three time points were set for data collection: baseline (preintervention), end of intervention (postintervention), and follow-up at 3 months after intervention (3 months postintervention).

### Instruments

ADAS-Cog scale: The Chinese version of ADAS-Cog scale (Chu et al., 2000) was applied to measure the severity of cognitive impairment, containing 12 items measuring multiple domains to assess the severity of cognitive impairment, with Cronbach’s α of 0.84. The total score on the ADAS-Cog obtained from all items ranges from 0 to 75, with higher scores revealing more severe cognitive dysfunction.

CSDD scale: The Chinese version of CSDD scale (Lin and Wang, 2008) was applied to evaluate signs and symptoms of depression for older adults with MCI or mild dementia and contains 19 items under 5 subscales (mood-related findings, physical findings, behavioral changes, cyclic functions, and cognitive changes), with Cronbach’s α of 0.87. Each item is scored between 0 (absent) and 2 (severe), and higher scores reveal greater depressive mood.

NPI: The Chinese version of NPI was applied to evaluate patients’ behavioral symptoms and their caregivers’ distress (Wang et al., 2012), which contains 12 categories of problem behavior. Each symptom is scored by multiplying severity (1–3) by frequency (1–4), yielding a maximum total score of 144. Caregiver distress is evaluated from 0 to 5. Higher scores indicate more severe symptoms and greater caregiver distress. The values of Cronbach’s α for symptom severity and caregiver distress were 0.83 and 0.81, respectively.

Spirituality Index of Well-Being (SIWB): The Chinese of the SIWB scale was applied to assess subjective wellbeing of the respondents (Lee and Salman, 2016). The scale contains 12 items among which items 1–7 belonging to a self-efficacy subscale and items 8–12 belonging to a life-scheme subscale, with each item scored from 1 (strongly agree) to 5 (strongly disagree). Higher scores reveal greater perceived spiritual wellbeing. The scale shows Cronbach’s α of 0.86.

### Statistical analysis

The obtained data were statistically processed using IBM SPSS Statistics 27.0 for Windows (IBM, Armonk, NY, USA). A *p*-value of < 0.05 (two tailed) was used to denote significant difference. Quantitative data normally distributed are presented as a mean value with a standard deviation (SD) and analyzed by using the independent *t*-test. The repeated measures analysis of variance (ANOVA) was performed for post-treatment and follow-up results to assess the main and interaction effects.

## Results

### Demographic and clinical characteristics of participants

**FIGURE 1:**
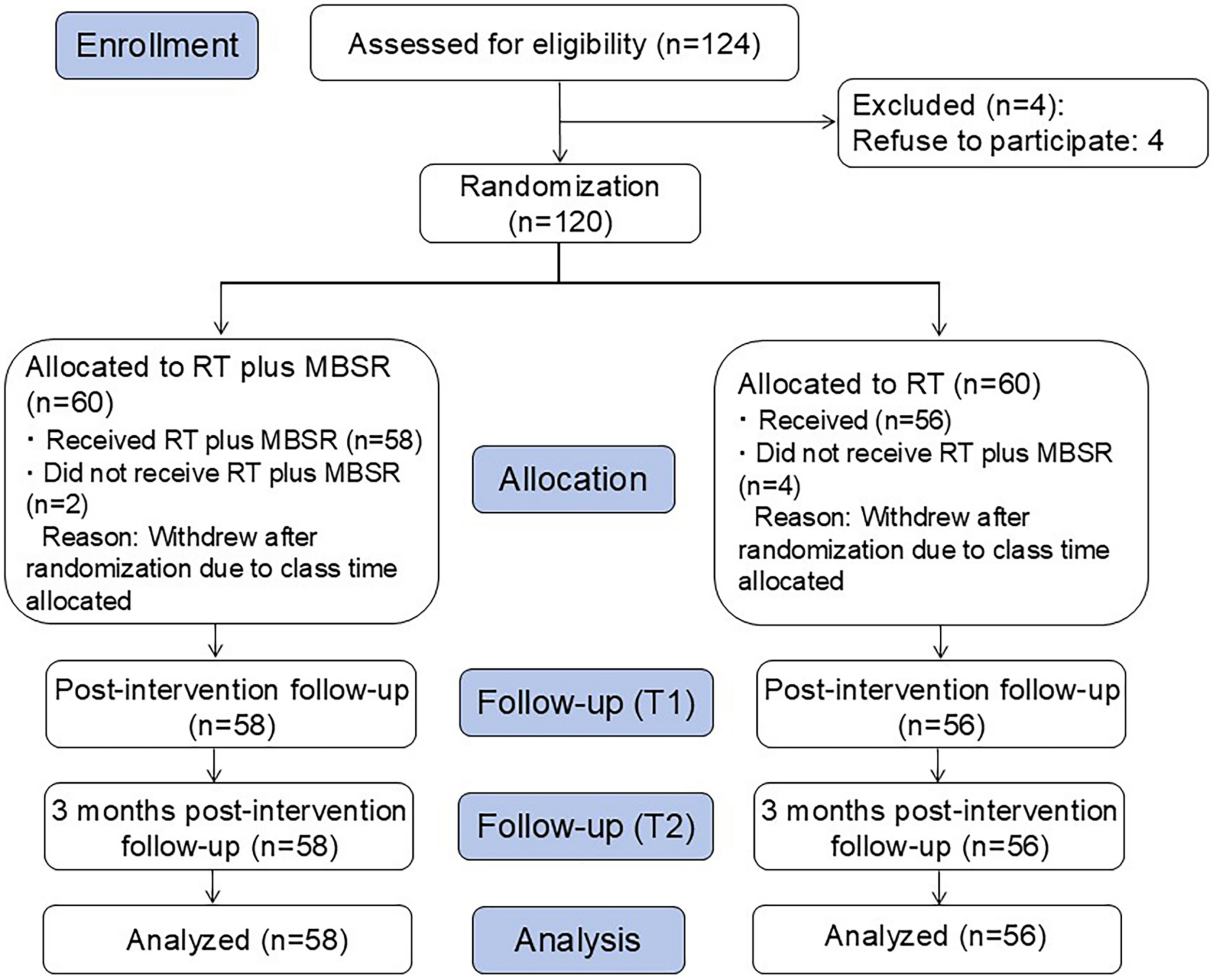
Consolidated Standards of Reporting Trials (CONSORT) flow diagram. MBSR, mindfulness-based stress reduction; RT, reminiscence therapy.

A total of 124 patients with MCI or mild dementia were initially selected for this study, and 4 were excluded due to their refusal to participate in the study. Thus, 120 patients were assigned into RT+MBSR group and RT group in equal numbers with 1:1 allocation. Two participants from the RT+MBSR group and 4 participants from the RT group withdrew from the study after randomization and thus leaving 58 patients in the RT+MBSR group and 56 patients in the RT group (Figure 1). Demographic data of participants between RT+MBSR group and RT group are listed in Table 1. No significance was noted in age, sex distribution, educational level, residence, proportion of MCI or mild dementia between RT+MBSR group and RT group.

**TABLE 1:** Demographic characteristics of participants in the RT+MBSR group and the RT group.

Characteristics	RT+MBSR group (*n* = 58)	RT group (*n* = 56)	*P*
Age (years), mean (SD)	76.5 (5.2)	77.7 (5.4)	0.236
Sex distribution, *n* (%)			0.850
Male	34 (58.6%)	31 (55.4%)	
Female	24 (41.4%)	25 (44.6%)	
Educational level, *n* (%)			0.847
Primary education	37 (63.8%)	34 (60.7%)	
Secondary education and above	21 (36.2)	22 (39.3%)	
Marital status, *n* (%)			0.442
Married	38 (65.5%)	32 (57.1%)	
Divorced or widowed	20 (34.5%)	24 (42.9%)	
Residence, *n* (%)			0.493
Urban	44 (75.9%)	46 (82.1%)	
Rural	14 (24.1%)	10 (17.9%)	
MCI or mild dementia, *n* (%)			1.000
MCI	29 (50.0%)	29 (51.8%)	
Mild dementia	29 (50.0%)	27 (48.2%)	

MBSR, mindfulness-based stress reduction; RT, reminiscence therapy; MCI, mild cognitive impairment; Quantitative data are presented as mean ± SD and analyzed by using the unpaired *t*-test. Count data are analyzed by using the chi-squared tests.

### Post-intervention differences in patient’s cognitive function

The cognitive function of patients was evaluated by the ADAS-Cog scale (Table 2). The repeated measures ANOVA of the ADAS-Cog scale scores revealed an interaction between group and time (*P* = 0.022). Further group analysis demonstrated lower ADAS-Cog scale scores in the RT+MBSR group than the RT group post intervention (*P* = 0.012; mean change: −3.5; 95% CI: −6.4 to −0.6) and 3 months post intervention (*P* = 0.007; mean change: −3.7; 95% CI: −6.6 to −0.8). The two groups did not significantly differ on the ADAS-Cog scale scores at baseline (*P* > 0.05). The ADAS-Cog scale scores showed significant improvements from preintervention to post intervention (*P* < 0.001) and from preintervention to 3 months post intervention (*P* = 0.006) in the RT+MBSR group but did not in the RT group.

**TABLE 2:** Post-intervention differences in patient’s cognitive function between the RT+MBSR group and the RT group.

Time point	RT+MBSR group (*n* = 58)	RT group (*n* = 56)
Preintervention, mean (SD)	27.3 (6.9)	26.8 (6.5)
Post intervention, mean (SD)	22.4 (5.3)	25.9 (6.3)
3 months post intervention, mean (SD)	23.5 (5.6)	27.2 (7.8)
Pre to post, mean diff. (95% CI)	4.9 (2.0 to 7.8)*	0.9 (−2.1 to 3.9)
Pre to 3 months post, mean diff. (95% CI)	3.8 (0.9 to 6.7)*	−0.4 (−3.4 to 2.6)
Post to 3 months post, mean diff. (95% CI)	−1.1 (−4.0 to 1.8)	−1.3 (−4.3 to 1.7)

MBSR, mindfulness-based stress reduction; RT, reminiscence therapy; CI, confidence interval; *P < 0.05 indicates significant time effects by the repeated measures ANOVA.

### Post-intervention differences in patient’s depression symptoms

The depressive symptoms of patients were evaluated by the CSDD scale (Table 3). The repeated measures ANOVA of the CSDD scale scores suggested an interaction between group and time (*P* = 0.031). Further group analysis demonstrated lower CSDD scale scores in the RT+MBSR group than the RT group post intervention (*P* < 0.001; mean change: −2.3; 95% CI: −3.7 to −0.9) and 3 months post intervention (*P* = 0.003; mean change: −2.0; 95% CI: −3.4 to −0.6). The two groups did not significantly differ on the CSDD scale scores at baseline (*P* > 0.05). There were significant improvements in the CSDD scale scores from preintervention to post intervention (*P* < 0.0001) and from preintervention to 3 months post intervention (*P* < 0.0001) in RT+MBSR group. For the RT group, the improvement of CSDD scale scores was only found from preintervention to post intervention (*P* = 0.048).

**TABLE 3:** Post-intervention differences in patient’s depressive symptoms between the RT+MBSR group and the RT group.

Time point	RT+MBSR group (*n* = 58)	RT group (*n* = 56)
Preintervention, mean (SD)	7.5 (3.9)	7.8 (3.8)
Post intervention, mean (SD)	4.1 (2.0)	6.4 (2.5)
3 months post intervention, mean (SD)	5.1 (2.1)	7.1 (3.5)
Pre to post, mean diff. (95% CI)	3.4 (2.0 to 4.8)*	1.4 (−0.01 to 2.8)*
Pre to 3 months post, mean diff. (95% CI)	2.4 (1.0 to 3.8)*	0.7 (−0.7 to 2.1)
Post to 3 months post, mean diff. (95% CI)	−1.0 (−2.4 to 0.4)	−0.7 (−2.1 to 0.7)

MBSR, mindfulness-based stress reduction; RT, reminiscence therapy; CI, confidence interval; *P < 0.05 indicates significant time effects by the repeated measures ANOVA.

### Post-intervention differences in patient’s behavioral symptoms

Patients’ behavioral symptoms and their caregivers’ distress were assessed by the NPI (Table 4). Significant group-by-time interactions were noted by the repeated measures ANOVA in both severity scores (*P* = 0.032) and caregivers’ distress scores (*P* = 0.022). The RT+MBSR group exhibited lower severity scores than the RT group post intervention (*P* = 0.002; mean change: −6.8; 95% CI: −11.6 to −2.1) and 3 months post intervention (*P* < 0.001; mean change: −7.5; 95% CI: −12.3 to −2.8). Similarly, lower caregivers’ distress scores were observed in the RT+MBSR group than the RT group post intervention (*P* = 0.018; mean change: −2.8; 95% CI: −5.3 to −0.4) and 3 months post intervention (*P* = 0.024; mean change: −2.7; 95% CI: −5.1 to −0.3). The two groups did not significantly differ on severity scores or caregivers’ distress scores at baseline (*P* > 0.05). The severity score showed significant improvements from preintervention to post intervention (*P* < 0.0001) and from preintervention to 3 months post intervention (*P* < 0.0001) in both RT+MBSR group and RT group. The caregivers’ distress scores exhibited significant improvements from preintervention to post intervention (*P* < 0.0001) and from preintervention to 3 months post intervention (*P* < 0.001) in the RT+MBSR group but did not in the RT group.

**TABLE 4:** Post-intervention differences in patient’s behavioral symptoms between the RT+MBSR group and the RT group.

Time point	RT+MBSR group (*n* = 58)	RT group (*n* = 56)
Severity symptom		
Preintervention, mean (SD)	43.4 (10.2)	44.2 (10.4)
Post intervention, mean (SD)	26.8 (11.3)	33.6 (10.5)
3 months post intervention, mean (SD)	23.3 (10.2)	30.8 (10.7)
Pre to post, mean diff. (95% CI)	16.6 (11.9 to 21.3)*	10.6 (5.8 to 15.4)*
Pre to 3 months post, mean diff. (95% CI)	20.1 (15.4 to 24.8)*	13.4 (8.6 to 18.2)*
Post to 3 months post, mean diff. (95% CI)	3.5 (−1.2 to 8.2)	2.8 (−2.0 to 7.6)
Caregivers’ distress		
Preintervention, mean (SD)	17.5 (5.4)	16.8 (6.0)
Post intervention, mean (SD)	11.8 (4.7)	14.6 (5.2)
3 months post intervention, mean (SD)	13.6 (5.2)	16.3 (5.8)
Pre to post, mean diff. (95% CI)	5.7 (3.3 to 8.1)*	2.2 (−0.25 to 4.6)
Pre to 3 months post, mean diff. (95% CI)	3.9 (1.5 to 6.3)*	0.5 (−1.9 to 2.9)
Post to 3 months post, mean diff. (95% CI)	−1.8 (−4.2 to 0.6)	−1.7 (−4.1 to 0.7)

MBSR, mindfulness-based stress reduction; RT, reminiscence therapy; SD, standard deviation; CI, confidence interval; *P < 0.05 indicates significant time effects by the repeated measures ANOVA.

### Post-intervention differences in patient’s spiritual wellbeing

Patients’ spiritual wellbeing was assessed by the SIWB scale (Table 5). The group time interaction, performed by the repeated measures ANOVA, was significant for the SIWB scale scores (*P* = 0.003), which revealed a significant increase of perceived spiritual wellbeing in the RT+MBSR group compared to the RT group post intervention (*P* < 0.001; mean change: 6.6; 95% CI: 3.5 to 9.7) and 3 months post intervention (*P* = 0.005; mean change: 5.1; 95% CI: 2.0 to 8.2). The two groups did not significantly differ on the SIWB scale scores at baseline (*P* > 0.05). The RT+MBSR group presented significant improvements of SIWB scale scores from preintervention to post intervention (*P* < 0.0001) and from preintervention to 3 months post intervention (*P* < 0.0001) but the RT group did not.

**TABLE 5:** Post-intervention differences in patient’s spiritual wellbeing between the RT+MBSR group and the RT group.

Time point	RT+MBSR group (*n* = 58)	RT group (*n* = 56)
Preintervention, mean (SD)	35.4 (6.2)	34.8 (6.4)
Post intervention, mean (SD)	43.8 (7.4)	37.2 (7.1)
3 months post intervention, mean (SD)	41.1 (7.5)	36.0 (6.8)
Pre to post, mean diff. (95% CI)	−8.4 (−11.5 to −5.3)*	−2.4 (−5.5 to 0.7)
Pre to 3 months post, mean diff. (95% CI)	−5.7 (−8.8 to −2.6)*	−1.2 (−4.3 to 1.9)
Post to 3 months post, mean diff. (95% CI)	2.7 (−0.4 to 5.8)	1.2 (−1.9 to 4.3)

*P < 0.05 indicates significant time effects by the repeated measures ANOVA.

## Discussion

In the present study, we examined the preliminary effectiveness of the MBSR program in combination with RT, each for 8 weeks, in older adults mildly-impaired by dementia. The main findings indicate that this combination could improve cognitive function, depressive, behavioral symptoms, and spiritual wellbeing of older adults living with early dementia both immediately and 3 months after the intervention.

Although RT has been widely used in a dementia care setting, comprehensive analysis yielded inconsistent findings regarding the effects of RT due to different settings (care home and community) and modalities (group and individual) (Macleod et al., 2021). Care home studies have shown several benefits on older people thriving with dementia from RT in the domains of cognition function, psychological wellbeing, quality of life, and communication (Lok et al., 2019; Yanagida et al., 2024b). A pilot randomized controlled trial demonstrated applying RT at home could improve cognitive function and reduce depression for community-dwelling older adults with mild dementia (Jung and Kwon, 2025). Individual RT is associated with possible benefits for cognition and quality of life (Perez-Saez et al., 2022). Group RT is associated with probable improvements in depressive symptoms and meaning of life in community-dwelling older adults (Zeleníková et al., 2025). In this study, group RT was performed for older adults living with MCI or mild dementia, and improvements on patients’ depressive symptoms and behavioral symptoms were noted after RT intervention.

MBSR can systematically teach participants to understand the principles of self-regulation by mindfulness, develop skill and autonomy in mindfulness practice (Tran et al., 2023). The MBSR was endorsed to be used by caregivers and psychotherapists of nursing homes in a bid to improve the physical and mental condition of older population (Javadzade et al., 2024). Mindfulness protocols produced an improvement in memory and attention. In a therapeutic context, life-storytelling, reminiscence and mindfulness are implemented to support the wellbeing of people with dementia (Niedderer et al., 2022). Dispositional mindfulness was found to be associated with the risk of anxiety and depressive symptoms in older adults with neurocognitive disorders, suggesting high mindful awareness may reduce the adverse effects of chronic physical morbidity on mental health (Lam et al., 2021). Reitano et al. (2023) developed a new integrated and multidisciplinary cognitive rehabilitation program involving mindfulness and reminiscence activities in patients with Parkinson’s disease and mild cognitive impairment. They demonstrated the effectiveness of this cognitive rehabilitation program in improving cognitive function and reducing depression symptoms. As shown by our results, older patients showed an improved cognitive function after receiving a combination of MBSR and RT compared to those receiving RT alone. For MCI, a change of +2 to +3 points on the ADAS-Cog was considered as minimal clinically important differences (MCIDs), and for mild AD, a change of +3 points on the ADAS-Cog was considered as MCIDs. In our study, a change of +4.9 points on the ADAS-Cog scores from baseline to post-intervention with a change of +3.8 points from baseline to 3 months post intervention in the RT+MBSR group were found, which met MCIDs. Accordingly, we examined the effects of a combination of MBSR and RT in improving the BPSD and mental health of older adults with MCI or mild dementia. Obtained data showed that this combination effectively improved depressive and behavioral symptoms of older adults living with MCI or mild dementia immediately. These improvements can sustain for 3 months after the intervention, compared to RT alone. The MCIDs of NPI ranges were 2.77 to 3.18 for severity and 3.10 to 3.95 for distress (Mao et al., 2015). In our study, the changes of NPI from baseline to post-intervention and 3 months post intervention met MCIDs in both groups. Possibly, RT only improves BPSD and helps for highly agitated or depressed patients or patients at certain times of day (sundowning syndrome), and its combination with MBSR adds additional effects on cognitive functioning and depressive symptoms. In addition to the currently used quality of life measures, adequate measures of momentary subjective wellbeing are also important in clinical dementia trials (Miklitz et al., 2024). Schlosser et al. (2023) reported only limited effects of a mindfulness-based intervention on psychological wellbeing in older adults with subjective cognitive decline. After combining MBSR with RT, our study demonstrated an improvement in spiritual wellbeing of older adults with MCI or mild dementia both immediately after the intervention and 3 months after the intervention.

This study has several limitations. First, there is theoretical potential for enhanced reporting of subjective outcomes due to lack of blinding for caregivers and patients to be aware of their treatment group as a substantial time commitment imbalance had created between two groups. Future studies should focus on having more objective scales, such as the Montreal Cognitive Assessment scale, in addition to self-report measures. Second, we did not formally assess participants’ expectations of mindfulness-based therapy before study enrollment. Expectation or beliefs about mindfulness may influence the efficacy of the intervention. Although a recent study demonstrated that neither patient expectancy nor perceived credibility of MBSR affected the efficacy of the intervention on improving cognition (Haddad et al., 2020). Further studies should examine the mindfulness level before study enrollment. Third, only 3-month follow-up data were obtained. Considering great psychological challenges and perceived stress of patients with dementia and their caregivers, a longer follow-up, such as 12 months after intervention is warranted to assess the sustainability of benefits of MBSR program in conjunction with RT effects over time. Forth, the sample size was relatively small and the study was performed in a single center, which limited the generalizability of MBSR program in conjunction with RT effects. Further external validity via multi-center studies is warranted.

In conclusion, the findings obtained from this study demonstrate that MBSR combined with RT is a feasible, effective non-pharmacological approach to improve cognitive function, depressive, behavioral symptoms, and spiritual wellbeing of older adults with MCI or mild dementia. This study explores a novel and safe combination to supporting dementia or MCI care practice for older adults in geriatric care units.

## Data Availability

The original contributions presented in this study are included in this article/supplementary material, further inquiries can be directed to the corresponding author.
